# It Takes Two to Tango: Links Between Traditional Beliefs About both Men’s and Women’s Gender Roles and Comfort Initiating Sex and Comfort Refusing Sex

**DOI:** 10.1007/s11199-023-01366-w

**Published:** 2023-04-29

**Authors:** Auguste G. Harrington, Jessica A. Maxwell

**Affiliations:** 1 Melbourne School of Psychological Sciences, University of Melbourne, Melbourne, 3010 Australia; 2 McMaster University, Hamilton, Canada

**Keywords:** Gender roles, Gender ideology, Sexual assertiveness, Sexual refusal, Sexual initiation

## Abstract

Traditional gender roles dictate rigid rules and standards prescribing which behaviors, thoughts, and feelings are considered masculine and feminine within sexual contexts, and thus internalizing these beliefs (higher traditional gender ideology) may influence sexual attitudes. Prior theorizing has primarily focused on how women’s traditional beliefs about women’s gender roles (traditional femininity ideology) and men’s traditional beliefs about men’s gender roles (traditional masculinity ideology) influence their sexual assertiveness. Yet, men can hold traditional beliefs about women, and women can hold traditional beliefs about men, and these beliefs should have important implications for sexual assertiveness. We addressed this gap by testing how both heterosexual women’s (*n* = 389) and men’s (*n* = 393) traditional masculinity and femininity ideologies associate with their reported comfort initiating sex and comfort refusing sex in their relationships. When accounting for both sets of beliefs, women’s traditional beliefs about men’s and women’s roles interacted to predict comfort initiating sex, but not comfort refusing sex. Men’s traditional beliefs about men’s roles predicted less comfort refusing sex, and their traditional beliefs about women’s roles predicted less comfort initiating sex. This novel research underscores the importance of considering beliefs about both sets of traditional gender roles for understanding people’s sexual attitudes.

**Supplementary Information:**

The online version contains supplementary material available at 10.1007/s11199-023-01366-w.

Traditional gender roles dictate which behaviors, thoughts, and feelings are considered masculine and feminine. Current perspectives in the literature primarily focus on how people’s beliefs about their own gender roles (e.g., a woman’s beliefs about how women should act) relate to relevant outcomes. Yet, people’s beliefs about the *other* set of traditional gender roles (e.g., a woman’s beliefs about how men should act) should also have important implications for their thoughts, feelings, and behavior. The influence of these beliefs is likely to be particularly evident in the sexual domain, where traditional gender roles prescribe strict and distinct expectations for men and women (e.g., “A woman should not initiate sex,” “A man should not turn down sex.”; Levant et al., 1992, 2007; Mahalik et al., 2005). Indeed, in monogamous mixed-sex sexual relationships, people’s attitudes should be influenced both by how they believe they should act as well as how they believe their partner should act, underscoring the need to account for people’s traditional beliefs about both men and women together. For instance, regardless of her beliefs about how women should act, a woman’s comfort initiating sex may be shaped by her beliefs that men should be sexually dominant and assertive. The current research illustrates the importance of considering men’s and women’s beliefs about *both* traditional gender roles by examining the links between these beliefs and attitudes towards initiating and refusing sex with their intimate partner.

## Traditional Gender Ideology: Limitations of Current Approaches

People internalize traditional masculine and feminine gender roles through social observation, and are influenced to behave in ways consistent with them through social reward and punishment (Bem, 1981, 1983; Levant, 1992, 1996, 2011; Levant & Richmond, 2016; Rudman & Fairchild, 2004). Individuals who have more strongly internalized traditional gender roles are higher in *traditional gender ideology* (Levant, 1996, 2011; Pleck, 1995; Thompson & Pleck, 1995), meaning they have more traditional beliefs regarding how men and/or women should act (more traditional beliefs about men’s and women’s roles). Traditional masculinity ideology (TMI) captures one’s internalization of masculine gender roles (traditional beliefs about how men should act), whereas traditional femininity ideology (TFI) captures one’s internalization of feminine gender roles (traditional beliefs about how women should act; Levant, 2011; Levant et al., 2007; Pleck, 1995).

Notably, because traditional gender ideology simply captures beliefs, men can endorse TFI (which captures their beliefs about how women should act), and women can endorse TMI (which captures their beliefs about how men should act). Indeed, men endorse TFI to a greater extent than women (Levant et al., 2007). Moreover, women’s TMI has implications for their attitudes, correlating with greater conservative political ideology, hostile sexism, benevolent sexism, and rape myth acceptance (Gerdes et al., 2018; McDermott et al., 2019, 2021). Despite the important theoretical implications of men’s TFI and women’s TMI, and even calls for investigations into this topic (Levant, 2011), prior theory and research have focused on the outcomes of people’s beliefs about *their own* gender roles, largely neglecting the consequences of beliefs about the *other* set of gender roles.

In addition to the lack of studies examining the effects of women’s TMI and men’s TFI, no prior research has simultaneously accounted for the effects of both traditional gender ideologies *together*, providing a limited picture of how these beliefs translate to key outcomes. As TMI and TFI represent distinct but closely related constructs (Levant, 2011; Levant et al., 2007), it is necessary to account for the effects of both simultaneously to fully understand what unique variance (if any) each predicts. For instance, without accounting for both traditional gender ideologies at once, we cannot conclude which predicts an outcome, if they interact, or if both predict an outcome independently.

To illustrate the importance of examining both men’s and women’s TMI and TFI, the current research will examine outcomes of these beliefs within a particularly relevant domain: men’s and women’s feelings of comfort initiating sex and comfort refusing sex with their romantic partner in committed, monogamous, mixed-sex relationships. Accounting for people’s endorsement of both sets of traditional beliefs within this domain fills a critical gap in the literature because (1) there is considerable theoretical rationale to support the idea that people’s beliefs about what behaviors, thoughts, and feelings to expect from their partner’s gender roles in situations related to sex may have a significant impact on their own attitudes and behaviors (Gerdes et al., 2018; Levant, 2011; Levant et al., 2007; McDermott et al., 2019), and (2) these rationales are distinct from those pertaining to beliefs about the person’s own gender roles.

## Traditional Gender Ideology and Sexual Initiation and Refusal

The rigid set of constraints traditional gender roles establish concerning sex dictate specific expectations for men’s and women’s sexual assertiveness (Levant et al., 2007; Mahalik et al., 2005). Sexual assertiveness—one’s comfort and ability to initiate and refuse sexual activity as well as negotiate desired sexual outcomes (Morokoff et al., 1997)—is essential for achieving desired intimacy within intimate relationships. Although exact definitions and subcomponents of sexual assertiveness vary (see discussion by Loshek & Terrell 2015), the construct is a key predictor of important behaviors like condom use (Stoner et al., 2008) and outcomes such as sexual and relationship satisfaction (Greene & Faulkner, 2005; Hurlbert, 1991; Morokoff et al., 1997). When examining the influence of traditional gender ideology, sexual assertiveness may be preferable to assessing enacted sexual behavior because people are often forced to behave in ways conflicting with gender role expectations. For instance, women must sometimes behave assertively, such as when negotiating for a salary, and men must sometimes be dependent on others, such as when they are in committed, intimate relationships (see the wide body of research examining gender role discrepancy strain, e.g., Levant & Powell, 2017). People may also engage in sex for many reasons (see review by Muise & Impett, 2016). Assessing sexual assertiveness rather than sexual behavior circumvents the influence of these complex contextual factors. We focus specifically on two aspects of sexual assertiveness that are common across popular conceptualizations and measures (e.g., The Sexual Assertiveness Scale; Morokoff et al., 1997; The Hurlbert Index of Sexual Assertiveness; Hurlbert, 1991; The Sexual Assertiveness Questionnaire; Loshek & Terrell, 2015), and that traditional gender roles dictate specific expectations for: comfort initiating sex and comfort refusing sex. We subsequently refer to comfort initiating and refusing sex together as sexual assertiveness for ease of expression and clarity.

Traditional gender roles dictate that men and women should have different patterns of sexual assertiveness. People higher in TMI believe that men should be assertive, dominant, and have a high sex drive—often initiating sex but never refusing it (Byers, 1996; Levant et al., 1992). Whereas people higher in TFI believe that women should be passive, compliant, and have a low sex drive—never initiating sex but remaining receptive to their partner’s advances (Byers, 1996; Levant et al., 2007). Despite the strong theorized influence of traditional gender beliefs on sexual assertiveness, these links are relatively underexplored. Prior research suggests that women who have more traditional beliefs about women’s roles are less sexually assertive (assessed as a unitary construct capturing various aspects of sexual assertiveness such as comfort refusing sex, initiating sex, and discussing contraception with one’s partner; Curtin et al., 2011; Greene & Faulkner, 2005; Morokoff et al., 1997). However, no research has directly examined the association between men’s TMI and their sexual assertiveness (see Gerdes et al., 2018; Levant & Richmond, 2007 for reviews of prior work examining the outcomes of men’s TMI). Yet, the clear expectations of traditional masculine roles for men in sexual domains (e.g., “A man should always be ready for sex,” “Men should always like to have sex”; McDermott et al., 2019) should mean that internalizing these roles to a greater extent will influence men’s comfort initiating sex and comfort refusing sex. Although prior research has tended to assess sexual assertiveness as a unitary construct, traditional gender ideology is likely associated with components of sexual assertiveness for different reasons (as outlined in detail in the following section). Thus, when examining links with gendered beliefs, it is necessary to examine comfort initiating sex and comfort refusing sex as separate outcomes.

In keeping with the dominant approach in the traditional gender ideology literature, the few studies that have explored the associations between gendered beliefs and sexual assertiveness have focused only on how beliefs about one’s own gender roles—but not the other set of traditional gender roles—relate to sexual attitudes. Yet, as sex in romantic relationships typically involves both members of the dyad, and couple members are interdependent, each partner can influence the other’s experiences in the sexual domain (for discussion of dyadic effects in sex research, see Muise et al., 2018), and an individual’s perception of their partner’s gender roles should influence their comfort initiating sex and comfort refusing sex. Thus, it is imperative to account for both how an individual thinks they should behave and how they believe their partner will react to their behavior, such as a man’s belief that a woman will react to his sexual assertiveness with passive compliance. In the following section, we outline different predictions for how traditional beliefs about both men *and* women will relate to men’s and women’s comfort initiating and refusing sex with their intimate partner.

## Predictions

### Women’s Comfort Initiating Sex

Women’s TFI may undermine their ability to initiate sex with their romantic partners because traditional feminine gender roles dictate that women should have a low sex drive, be sexually reluctant, and have relatively few sexual needs (Byers, 1996), and internalization of these ideas may restrict women from initiating sex on their own behalf. Moreover, traditional feminine gender roles dictate that women be passive, compliant, and deferent (Byers, 1996; Levant et al., 2007), and these roles are inconsistent with traits needed to initiate sex (e.g., vocalizing one’s desires). However, women’s TMI should also undermine their comfort initiating sex. As traditional masculine gender roles dictate that men be dominant, active, and assertive initiators of sexual activity (Byers, 1996; Levant et al., 1992), women who hold these beliefs (i.e., are higher in TMI) may feel that depriving men of this role may threaten their masculinity, leading to negative consequences such as anger, hurt feelings, and even rejection (e.g., Lamarche et al., 2020). Moreover, women who believe that men are highly motivated to engage in sexual activity may view their partner’s initiation as a demonstration of their affection and a lack of initiation as a sign of problems within the relationship. Thus, by initiating sex themselves, women who hold traditional views of men may feel they risk reprisal and deprive themselves of a useful relationship diagnostic (i.e., gauging their partner’s affection).

### Women’s Comfort Refusing Sex

Like with sexual initiation, women’s TFI may undermine their ability to refuse sex because neglecting their partner’s ‘needs’ by refusing sex may violate traditional feminine roles dictating that women must nurture and care for their partner (Bay-Cheng & Eliseo-Arras, 2008; Gavey, 1992; Lewin, 1985; Russell, 1982; Small & Kerns, 1993). Women’s TFI may also undermine their ability to refuse sex because refusing sex can require forceful or repeated resistance, inconsistent with the passive, submissive, and unassertive nature of traditional femininity (Byers, 1996). However, women’s TMI may also undermine their sexual assertiveness through the belief that men’s sex drive is ‘unstoppable’ and that interruption of this momentum during intimacy may result in men getting ‘carried away’ (Gavey, 2005; Gavey et al., 2001; MacCorquodale, 1989; Miller & Marshall, 1987; Weiss, 2009). This view of men’s sexual behavior may lower women’s ability to refuse sex as it suggests that refusal may have negative consequences such as partner dissatisfaction or even pressure and coercion (Gavey, 2005; Katz & Tirone, 2010; Muehlenhard & Cook, 1988). Indeed, women may opt for unwanted sex with their romantic partner to avoid their partner’s dissatisfaction or continued pressure for sex (Basile, 1999; Gavey, 1992, 2005; Livingston et al., 2004), as well as because of indirect pressure from cultural norms (e.g., Gavey, 1992, 2005).

### Men’s Comfort Initiating Sex

Traditional masculine roles dictate that men should be agentic and dominant in sexual encounters, increasing the extent of sexual activities in any given intimate interaction and using assertiveness to overcome ‘token’ resistance from their female partners (Byers, 1996). Thus, men higher in TMI should feel especially empowered to initiate sex. However, men’s TFI should also increase their comfort initiating sex as traditional feminine roles dictate that women should nurture and care for their partner, even at the expense of their own needs (Byers, 1996). Thus, men who hold these beliefs may expect women to remain sexually available for their pleasure (Byers, 1996; Lewin, 1985; Russell, 1982), increasing their comfort initiating sex. Moreover, traditional feminine roles dictate that women mount ‘token resistance’ against sexual advances, gently limiting men’s sexual advances to maintain the appearance of ‘purity’ (Kim et al., 2007; MacCorquodale, 1989; Tolman et al., 2007) and men who hold these beliefs about women’s roles may feel justified in persisting in the face of legitimate sexual refusals. In support of this idea, men with more traditional views of women and women’s roles are more likely to perpetrate unwanted sex (Byers & Wilson, 1985; Koss et al., 1985, 1987; Rapaport & Burkhart, 1984; see Walker, 1997 for review).

### Men’s Comfort Refusing Sex

Traditional masculine roles dictate that men be highly motivated to engage in sexual activity and be willing to exploit or pursue any sexual opportunity made available by a woman (Byers, 1996). Therefore, even though the masculine sexual role is highly agentic, internalizing this role should undermine men’s ability to refuse sex (Fagen & Anderson, 2012; Murray, 2018). However, men higher in TFI who hold the traditional belief that women’s role is to nurture and support them may also feel unable to refuse sex—even if their sexual desire is low—as they may consider their partner’s initiation to be a form of caring for them or relationship maintenance. Further, men who hold traditional beliefs that women are delicate and must be protected may feel that refusing their partner’s sexual advances could hurt them (Murray, 2018). Finally, men who believe women have very low sex drives may feel pressured to accept sex because they may believe their partner’s sexual initiation to be too rare an opportunity to pass up.

## Overview of Current Research

In sum, the purpose of the current research was to examine how men’s and women’s traditional gender ideologies—including their views about their own gender roles, as well as their views about their partner’s gender roles—relate to their feelings of comfort initiating and refusing sex. Our novel approach involved examining both TMI and TFI together, accounting for their shared variance. However, to highlight the importance of adopting this approach, we first present the links (i.e., correlations) between women’s TFI and men’s TMI and sexual assertiveness in isolation (the most traditional approach), as well as women’s TMI and men’s TFI in isolation (a novel, but still limited approach). Then, to overcome the limitations of testing either set of beliefs in isolation, we model men’s and women’s TFI and TMI against each other as alternative predictors to determine what unique variance (if any) each predicts in comfort with sexual initiation and refusal and if any interactions emerge.

Our predictions above focus on the effects of TMI and TFI *within* each gender rather than differences across men and women. Although prior studies have validated measures of TMI and TFI for both women and men, we posit that the TMI and TFI constructs will not have equivalent meanings for men and women and thus assess different constructs between genders. For instance, for men, TMI relates to beliefs about their own gender and thus how they themselves should behave as a man, and we will test how these self-related expectations are linked to men’s own sexual assertiveness. By contrast, for women, TMI captures beliefs about men and thus how they think their partner should behave as a man. Supporting these theorized differences, previous research testing for invariance of TMI and TFI across gender have found mixed support for metric invariance (suggesting that the factor loadings of the items may not be invariant across genders) and scalar invariance (suggesting that difference in the latent means across gender could be due to structure differences in the latent variable; McDermott et al., 2017; Levant et al., 2013, 2017). In sum, because (1) the estimated effects represent either self-related or other-related expectations depending on the gender of the respondent (thus test different psychological processes) and (2) TMI and TFI are potentially not statistically equivalent across gender, we do not believe it is appropriate to directly compare the effects of traditional ideology between men and women. Thus, our tests of predictions will focus on examining the associations between TMI, TFI, and sexual assertiveness within men and women separately.

## Method

### Participants

As part of a broader study with diverse aims, we recruited 840 participants using Prolific, an online crowd-sourcing platform (https://www.prolific.co/; see Palan & Schitter, 2018). We collected the data just prior to the COVID-19 pandemic (end of February 2020). No findings have been published from this broader study to date. All participants completed identical measures for the current research, which were part of either a longer (40 min) or shorter (20 min) version of the broader study (see OSM for all measures used). We compensated participants with £4.17 GBP or £2.09 GBP, respectively.

The broader study eligibility criteria were sexually active people in committed mixed-sex romantic relationships over the age of 18 who were working or studying and did not have children. However, because these criteria were not of relevance to the current research, we retained participants even if they were not working/studying or had children. We excluded 30 participants who failed to pass the attention check(s) embedded in the survey (see OSM Sect. 3 for attention check items). As our focus is on how traditional beliefs influence attitudes in committed, mixed-sex, sexual relationships, for the present analyses, we further excluded 18 participants who did not identify as heterosexual, five who were in casual relationships, three who were unlikely to be having sex regularly with their partner (those on a relationship ‘break’ or in long-distance relationships), and two who did not identify as either men or women. Our remaining sample (*N* = 782) comprised 393 men and 389 women. Participants ranged in age from 18 to 76 (*SD* = 10.63) and had been in their relationships for an average of 7 years (*SD* = 7.30). The self-reported ethnicity of our participants was as follows (participants could select multiple ethnicities; thus, percentages sum greater than 100%): White/Caucasian (658, 84.1%), East Asian (50, 6.4%), Black/African-American (26, 3.3%), Latin American/Hispanic (23, 2.9%), South Asian (18, 2.3%), South East Asian (19, 2.4%), Pacific Islander (6, 0.8%), African (5, 0.6%), Indigenous/Aboriginal (4, 0.5%), Arab/West Asian (4, 0.5%), and ‘Other’ (13, 1.7%). The self-reported relationship status of our participants was as follows: Living together (36.2%), Married (28.5%), Serious (27.6%), Steady/Exclusively dating (6.9%), and Engaged (0.8%).

We conducted a conservative power analysis (Faul et al., 2009) that sought to account for the key differences we planned to examine. We aimed to have complete data from 400 men and 400 women to ensure adequate statistical power to detect a small effect size. Results of a sensitivity analysis for our original planned analytic approach suggest this sample size would allow us to detect a small effect (*f*^2^ = 0.02) with 0.95 power in a MANOVA analysis (to account for two correlated outcome variables: comfort initiating sex and comfort refusing sex) with two groups (i.e., men and women), and seven predictors. During the review process our analytic approach changed to a multiple regression within each gender. Nevertheless, this sample size of 400 per gender also affords 80% statistical power to detect small effects (*f*^2^ = 0.02) when examining a multiple regression analysis with three predictors within each gender. Traditional masculinity and femininity ideologies are correlated (in our sample: *r*(782) = 0.57, *p* < .001) but distinct constructs and therefore likely predict some of the same variance in sexual assertiveness. Thus, we aimed to achieve a high degree of statistical power to detect small differences in the predictive ability of these constructs.

### Procedure and Measures

The authors’ university ethics committee approved the procedure, which included additional measures beyond the scope of the present study. Participants first completed a measure of self-esteem, followed by measures of traditional masculinity and femininity ideology, comfort initiating sex, comfort refusing sex, sexual knowledge and skills, and finally demographic information (See OSM Sect. 2 for all items employed in the present study).

#### Traditional Masculinity Ideology

To assess traditional masculinity ideology, we administered The Male Role Norms Inventory Very Brief (MRNI-VB; McDermott et al., 2019), a five-item assessment developed from the 21-item Male Role Norms Inventory Short Form (MRNI-SF; Levant et al., 2013). Participants rated their agreement with a series of normative statements about how men “should” or “should not” think, feel, and behave, such as “A man should always be the boss” (1 = *strongly disagree*, 7 = *strongly agree*), with higher scores indicating greater endorsement of traditional beliefs about men. Research has provided evidence for the reliability of the MRNI-VB, as well as convergent and discriminant evidence for its validity, and the scale has demonstrated comparable predictive ability to the larger MRNI-SF (McDermott et al., 2019).

#### Traditional Femininity Ideology

To assess traditional femininity ideology, we analyzed four items from the Dependency/Deference subscale and four items from the Purity subscale of the Femininity Ideology Scale (FIS; Levant et al., 2007; see OSM Sect. 1 for details on item selection). We focused on these subscales because they were most relevant to women’s and men’s sexual beliefs and sexual behavior. Participants rated their agreement with a series of normative statements about how women “should” or “should not” think, feel, and behave, e.g., “A woman should not swear.” (1 = *strongly disagree*, 7 = *strongly agree*), with higher scores indicating greater endorsement of traditional beliefs about women. Evidence has established the construct validity (discriminant and convergent) and reliability of the original 45-item measure (Levant et al., 2007).

#### Comfort Initiating Sex and Comfort Refusing Sex

To assess comfort initiating sex and comfort refusing sex—two central aspects of sexual assertiveness—we asked participants to rate two items regarding their current romantic relationship: “I am comfortable refusing sex” and “I am comfortable initiating sex” (1 = *strongly disagree*, 7 = *strongly agree*). Such single-item assessments are appropriate when the construct being measured is specific and unambiguous (see Allen et al., 2022).

#### Additional Variables

**Self-Esteem.** To confirm that any observed associations with sexual assertiveness were due to differences in traditional gender ideology and not a general lack of sexual confidence stemming from low global evaluations of self-worth, participants completed the Rosenberg Self-Esteem Scale (Rosenberg, 1965), which includes 10 items (e.g., “I feel that I’m a person of worth, at least on an equal plane with others;” 1 = *strongly disagree*, 7 = *strongly agree*). This variable was used in an additional analysis to bolster the robustness of our results.

**Sexual Knowledge and Skills.** To assess participants’ perceptions of their sexual knowledge and skills, we asked participants to rate one item with reference to their current romantic relationship: “I have the knowledge and skills needed to have a satisfying sex life.” (1 = *strongly disagree*, 7 = *strongly agree*). This variable was explored in an additional post hoc analysis.

## Results

### Descriptive Statistics

Table 1 displays the descriptive statistics, reliabilities, and bivariate correlations across our primary measures. The means and standard deviations suggest that participants felt generally comfortable initiating and refusing sex with their partner, but there was variance in this comfort. Independent samples *t*-tests indicated that, relative to women, men reported significantly greater masculinity ideology, femininity ideology, and self-esteem, but significantly less comfort refusing sex. There were no significant gender differences in comfort initiating sex, age, or sexual knowledge and skills.

**Table 1:** Descriptive Statistics and Correlations Between Measures

	Descriptive Statistics						Correlations						
		Men		Women		Gender difference							
	α	*M*	*SD*	*M*	*SD*	*t*(df)	1	2	3	4	5	6	7
1. Femininity Ideology	0.82	1.69	0.89	1.45	0.71	-4.55 (743.61)***	-	0.600***	-0.157**	-0.172***	0.026	0.083	-0.085
2. Masculinity Ideology	0.86	2.95	1.43	1.99	1.19	-10.34 (765.12)***	0.528***	-	-0.118*	-0.152**	0.096	0.137**	-0.048
3. Comfort Initiating Sex	-	5.63	1.44	5.62	1.48	-0.26 (788)	-0.130*	0.010	-	0.359***	0.201***	0.016	0.557***
4. Comfort Refusing Sex	-	5.19	1.48	5.72	1.36	5.38 (788)***	-0.184***	-0.200***	0.216***	-	0.142**	-0.087	0.283***
5. Self-Esteem	0.93	4.89	1.22	4.72	1.18	-2.10 (788)***	-0.022	0.014	0.298***	0.100*	-	0.237***	0.243***
6. Age	-	33.00	10.66	31.62	10.57	-1.84 (288)	0.110*	0.056	-0.082	-0.101*	0.108*	-	0.027
7. Sexual Knowledge and Skills	-	5.58	1.21	5.39	1.44	1.93(754)	-0.178***	-0.012	0.597***	0.157**	0.259***	-0.033	-

*Note.* Men *n* = 393, women *n* = 389. All measures used scales ranging from 1–7. Correlations for women are presented above the diagonal, correlations for men are presented below. ***p < .001. **p < .01. *p < .05. All other values are not significant (*p* > .05).

### Examining Ideologies in Isolation

As depicted in Table 1, consistent with predictions, women’s endorsement of both TFI and TMI were associated with lower comfort initiating sex and refusing sex. Also as expected, men’s endorsement of both TMI and TFI were associated with lower comfort refusing sex. However, contrary to our predictions, men’s endorsement of TFI was associated with *lower* comfort initiating sex, and there was no association between TMI and comfort initiating sex. These bivariate correlations provide initial evidence for the importance of examining not only people’s beliefs about their own gender roles but also their beliefs about the other set of gender roles. However, without accounting for both sets of beliefs simultaneously, we cannot confirm whether only one ideology predicts sexual assertiveness, if both do independently, or if they combine to influence sexual assertiveness.

### Main Analyses

Our main analyses aimed to examine how both women’s and men’s TMI and TFI predicted their comfort initiating sex and comfort refusing sex when modeled as simultaneous predictors alongside their interaction. To test our hypotheses, for each gender we conducted two linear regression analyses in SPSS version 26 predicting (1) comfort initiating and (2) comfort refusing sex from TMI, TFI, and the interaction of TMI and TFI. We estimated the effects of TMI and TFI on sexual initiation and refusal for women and men separately, for a total of four multiple regressions. Although comfort initiating and refusing sex were correlated (*r*(782) = 0.28, *p* < .001), the rationale for our predictions differed for each, so we examined them as separate dependent variables.

#### Women

**Fig. 1:**
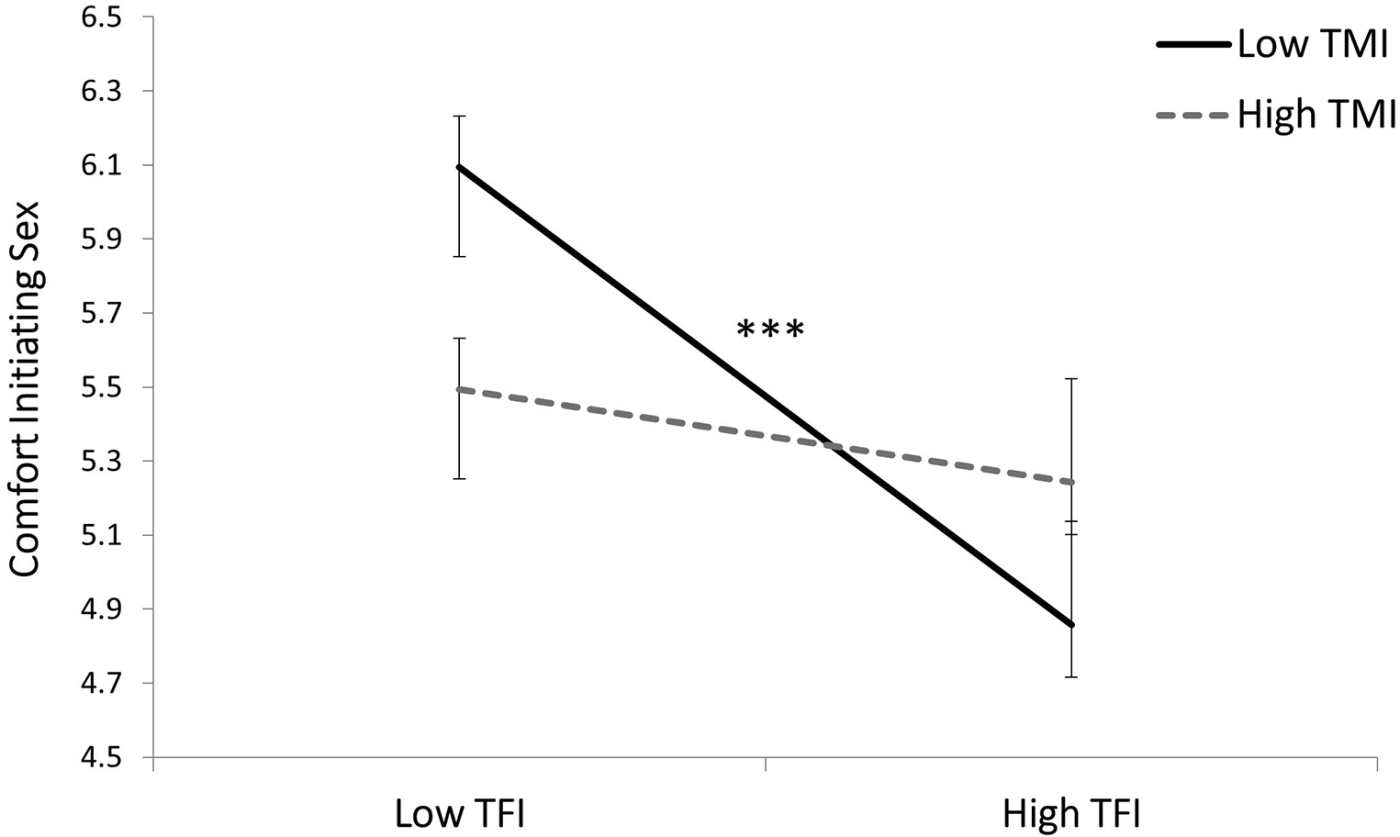
Women’s Comfort Initiating Sex as a Function of their Endorsement of Traditional Femininity Ideology (TFI) and Traditional Masculinity Ideology (TMI) *Note*. High and low values represent 1 *SD* above and below the mean. ***p < .001. All measures used scales ranging from 1 to 7.

Women’s greater TFI was not related to comfort refusing sex (see Table 2). No significant association emerged between women’s TMI and comfort refusing or initiating sex; however, a significant interaction emerged between women’s TMI and TFI predicting comfort initiating sex. This interaction is shown in Fig. 1, with slopes plotted at 1 *SD* above and below the sample mean. For women lower in TMI (-1 *SD*), higher TFI was associated with less comfort initiating sex (*β* = -0.75, *SE* = 0.22, *p* = .001, 95% CI [-1.86, -0.31]), but for women high in TMI (+ 1 *SD*), TFI was unassociated with comfort initiating sex (*β* = -0.15, *SE* = 0.14, *p* = .280, 95% CI [-0.42, 0.12]). Or, focusing on the effect of TMI across levels of TFI, for women low in TFI (-1 *SD*), greater endorsement of TMI was associated with less comfort initiating sex (*β* = -0.21, *SE* = 0.10, *p* = .034, 95% CI [-0.41, -0.02]), whereas for women high in TFI (+ 1 *SD*), greater TMI was not significantly associated with comfort initiating sex (*β* = 0.14, *SE* = 0.11, *p* = .191, 95% CI [-0.07, 0.33]). Taken together, comfort initiating sex was highest for women who showed low endorsement of both TFI and TMI.

**Table 2:** Regressions of Women’s Comfort Initiating Sex (Top Box) and Comfort Refusing Sex (Bottom Box) on Masculinity Ideology, Femininity Ideology, and their Interaction (Women Only)

Dependent Variable		*β*	*SE*	*t*	*p*	*95% CI*	
						Lower	Upper
Comfort Initiating Sex	**Femininity Ideology**	**-0.22**	**0.15**	**-3.09**	**<.001**	**-0.74**	**-0.16**
	Masculinity Ideology	-0.03	0.08	-0.50	.62	-0.19	0.11
	**Femininity x Masculinity Ideology**	**0.15**	**0.08**	**2.60**	**.01**	**0.05**	**0.38**
Comfort Refusing Sex	Femininity Ideology	-0.12	0.13	-1.72	.09	-0.50	0.03
	Masculinity Ideology	-0.08	0.07	-1.22	.22	-0.23	0.05
	Femininity x Masculinity Ideology	-0.01	0.08	-0.21	.83	-0.17	0.13

*Note*. *CI* = Confidence Interval. Women *n* = 389. All measures used scales ranging from 1–7. Predictor variables were mean-centered.

#### Men

Men’s greater TMI was related to greater comfort initiating sex, although this did not reach traditional levels of significance (*p* = .061), and was significantly related to lower comfort refusing sex (Table 3). Contrary to predictions, men’s greater endorsement of TFI was significantly associated with *less* comfort initiating sex, and there was no association between men’s TFI and comfort refusing sex (Table 3). No significant interaction effects between TMI and TFI emerged.

**Table 3:** Regressions of Men’s Comfort Initiating Sex (top box) and Comfort Refusing Sex (bottom box) on Masculinity Ideology, Femininity Ideology, and Their Interaction (men only)

Dependent Variable		*β*	*SE*	*t*	*p*	*95% CI*	
						Lower	Upper
Comfort Initiating sex	**Femininity Ideology**	**-0.17**	**0.12**	**-2.35**	**.02**	**-0.52**	**-0.05**
	Masculinity Ideology	0.11	0.06	1.88	.06	-0.01	0.23
	Femininity x Masculinity Ideology	-0.02	0.05	-0.31	.76	-0.12	0.09
Comfort Refusing Sex	Femininity Ideology	-0.10	0.12	-1.33	.18	-0.40	0.08
	**Masculinity Ideology**	**-0.14**	**0.06**	**-2.44**	**.02**	**-0.27**	**-0.03**
	Femininity x Masculinity Ideology	-0.02	0.05	-0.25	.81	-0.12	0.09

*Note*. *CI* = Confidence Interval. Men *n* = 393. All measures used scales ranging from 1–7. Predictor variables were mean-centered.

### Alternate Explanations

We wanted to ensure our effects presented in Tables 2 and 3 were robust to two possible confounds linked to our predictors and/or outcomes: self-esteem and age. Higher self-esteem captures general feelings of self-worth and may relate to greater sexual assertiveness (Ménard & Offman, 2009), and older age tends to relate to more traditional attitudes (Lynott & McCandless, 2000). We conducted two hierarchical regressions (one for comfort initiating sex, and one for comfort refusing sex) within each gender, entering grand-mean centered self-esteem and age at the first step and the grand-mean centered ideologies and their interaction in the second step (Tables 4 and 5). When all variables were included in the second step, for both men and women, greater self-esteem was associated with comfort initiating sex and comfort refusing sex. Age was associated with less comfort refusing sex for women and less comfort initiating and refusing sex for men. However, the significant associations between endorsement of TMI and TFI and sexual assertiveness reported in Tables 2 and 3 remained significant when accounting for age and self-esteem, and the ideologies explained a significant additional amount of variance in the outcomes. These additional analyses support that endorsing TMI and/or TFI is uniquely associated with sexual assertiveness, independent of negative self-views and age. Given concerns that negatively vs. positively worded items may assess different aspects of self-esteem (e.g., Gnambs & Schroeders 2020; Supple et al., 2013), we also re-conducted our control analyses using (a) only positively worded items, and (b) only negatively worded items from our measure of self-esteem. Observed effects were not significantly altered.

**Table 4:** Hierarchical Regressions of Women’s Comfort Initiating Sex (top box) and Comfort Refusing Sex (bottom box) on Age and Self-esteem, Masculinity Ideology, Femininity Ideology, and Their Interaction (women only)

Step	Dependent Variable		*β*	*SE*	*t*	*p*	*95% CI*			
							Lower	Upper	*R* ^*2*^	*∆R* ^*2*^
1	Comfort Initiating Sex								0.04	0.04***
		Age	-0.03	0.01	-0.658	.51	-0.02	0.01		
		**Self-Esteem**	**0.21**	**0.06**	**4.083**	**< .001**	**0.14**	**0.39**		
2									0.07	0.03***
		Age	-0.01	0.01	-0.158	.87	-0.02	0.01		
		**Self-Esteem**	**0.21**	**0.06**	**4.176**	**< .001**	**0.14**	**0.39**		
		**Femininity Ideology**	**-0.20**	**0.14**	**-2.968**	**.001**	**-0.71**	**-0.14**		
		Masculinity Ideology	-0.06	0.08	-0.919	.36	-0.22	0.08		
		**Masculinity Ideology x Femininity Ideology**	**0.15**	**0.08**	**2.578**	**.01**	**0.05**	**0.37**		
1	Comfort Refusing Sex								0.04	0.04***
		**Age**	**-0.13**	**0.01**	**-2.486**	**.01**	**-0.03**	**0.00**		
		**Self-Esteem**	**0.17**	**0.06**	**3.342**	**< .001**	**0.08**	**0.32**		
2									0.07	0.03**
		**Age**	**-0.11**	**0.01**	**-2.136**	**.03**	**-0.03**	**0.00**		
		**Self-Esteem**	**0.18**	**0.06**	**3.512**	**< .001**	**0.09**	**0.32**		
		Femininity Ideology	-0.11	0.13	-1.534	.13	-0.46	0.06		
		Masculinity Ideology	-0.08	0.07	-1.356	.18	-0.24	0.04		
		Masculinity Ideology x Femininity Ideology	-0.02	0.08	-0.389	.70	-0.18	0.12		

*Note*. *CI* = Confidence Interval. Women *n* = 389. Non-continuous measures used scales ranging from 1–7. Predictor variables were mean-centered. ***p < .001. **p < .01.

**Table 5:** Hierarchical Regressions of Men’s Comfort Initiating Sex (top box) and Comfort Refusing Sex (bottom box) on Age and Self-esteem, Masculinity Ideology, Femininity Ideology, and Their Interaction (men only)

Step	Dependent Variable		*β*	*SE*	*t*	*p*	*95% CI*			
							Lower	Upper	*R* ^*2*^	*∆R* ^*2*^
1	Comfort Initiating Sex								0.10	0.10***
		**Age**	**-0.12**	**0.01**	**-2.4**	**.02**	**-0.03**	**0.00**		
		**Self-Esteem**	**0.31**	**0.06**	**6.435**	**< .001**	**0.26**	**0.48**		
2									0.12	0.02*
		**Age**	**-0.10**	**0.01**	**-2.148**	**.03**	**-0.03**	**0.00**		
		**Self-Esteem**	**0.30**	**0.06**	**6.347**	**< .001**	**0.25**	**0.47**		
		**Femininity Ideology**	**-0.14**	**0.12**	**-2.032**	**.04**	**-0.46**	**-0.01**		
		Masculinity Ideology	0.10	0.06	1.779	.08	-0.01	0.21		
		Masculinity Ideology x Femininity Ideology	-0.03	0.05	-0.469	.64	-0.12	0.08		
1	Comfort Refusing Sex								0.02	0.02*
		**Age**	**-0.11**	**0.01**	**-2.243**	**.03**	**-0.03**	**0.00**		
		**Self-Esteem**	**0.11**	**0.06**	**2.227**	**.03**	**0.02**	**0.26**		
2									0.07	0.05***
		**Age**	**-0.10**	**0.01**	**-1.912**	**.06**	**-0.03**	**0.00**		
		**Self-Esteem**	**0.11**	**0.06**	**2.235**	**.03**	**0.02**	**0.25**		
		Femininity Ideology	-0.08	0.12	-1.064	.29	-0.37	0.11		
		**Masculinity Ideology**	**-0.15**	**0.06**	**-2.519**	**.01**	**-0.27**	**-0.03**		
		Masculinity Ideology x Femininity Ideology	-0.02	0.05	-0.349	.73	-0.12	0.09		

*Note*. *CI* = Confidence Interval. Men *n* = 393. Non-continuous measures used scales ranging from 1–7. Predictor variables were mean-centered. ***p < .001. *p < .05.

### Post Hoc Additional Analysis

**Fig. 2:**
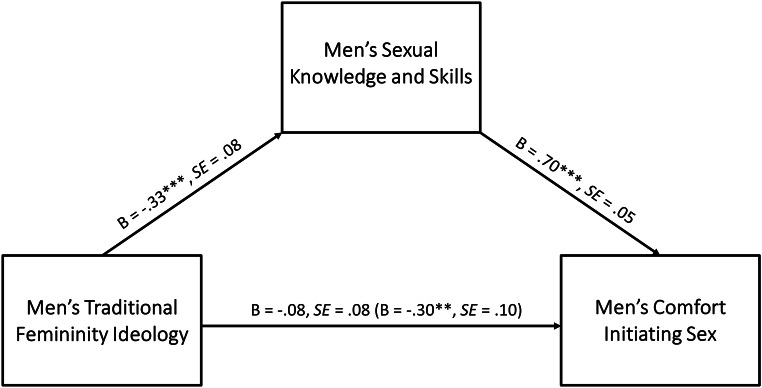
Indirect Effect of Men’s Greater TFI on Lower Comfort Initiating Sex Through Lower Sexual Knowledge and Skills Indirect effect: -0.23, *SE* = 0.07, 95% CI [-0.37, -0.09] *Note*. Estimates on the bottom path represent (1) the direct effect when accounting for the mediator and (2) the total effect (in parentheses). All measures used scales ranging from 1 to 7 and estimates are unstandardized. ***p < .001. **p < .01. *p < .05. All other values are not significant (*p* > .05).

In contrast to our prediction, our results suggested that men with more traditional beliefs about women (higher TFI) were *less* comfortable initiating sex. In an effort to understand this unexpected association, we reasoned that men who strongly endorse TFI (and thus view women as pure and non-sexual) may view sex and sexuality as taboo, and have less sexual knowledge and less sexual skills, ultimately leading to lower self-efficacy and decreased comfort initiating sex. To test this exploratory theorizing, we conducted a mediation analysis on only the men sampled using the PROCESS Macro for SPSS 26.0 (Model 4, estimating 10,000 bootstrap resamples; Hayes, 2017), testing whether the association between men’s TFI and comfort initiating sex was mediated by their perception of their sexual skills and knowledge about sex, while simultaneously accounting their TMI as a covariate. The results were consistent with our reasoning (see Fig. 2 for all estimates): men’s greater endorsement of TFI was associated with lower sexual knowledge and skills which, in turn, was associated with lower comfort initiating sex and the indirect effect of TFI on comfort initiating sex through knowledge and skills was significant. Moreover, accounting for the indirect effect reduced the direct effect of men’s TFI on comfort initiating sex below the threshold of significance. This analysis provided preliminary support for our reasoning that men who more strongly endorsed TFI feel less confident in their sexual knowledge and abilities, decreasing their comfort initiating sex.

## Discussion

The present study demonstrates how people’s traditional beliefs about both their own gender roles and the other set of traditional gender roles have critical implications for their attitudes, underscoring the importance of going beyond the dominant approach in the literature by examining the combination of both sets beliefs. We do so by illustrating how both men’s and women’s TMI and TFI are associated with important sexual attitudes within mixed-sex monogamous relationships, a theoretically relevant context wherein people’s attitudes should be influenced both by how they believe *they* should act and how they believe *their partner* should act. For women, we found that traditional beliefs about men and women interacted to predict their comfort initiating sex. For men, more traditional beliefs about women were related to less comfort initiating sex, and more traditional beliefs about men were related to less comfort refusing sex. Moreover, further highlighting the limitations of assuming that only one’s beliefs about their own gender are critical, some associations that emerged when considering each ideology in isolation were reduced to non-significance when controlling for both sets of beliefs. Thus, if we had not examined both sets of beliefs together, the results would have provided an incomplete picture. Future research exploring how traditional gender ideology relates to sexual attitudes should examine participants’ beliefs about both men and women to avoid misleading results. We elaborate below on how the current research expands our understanding of traditional gender ideology and sexual processes.

### Interpretation of Results

#### Women’s Traditional Beliefs

The results for women both replicate and extend prior research. When examined in isolation (i.e., bivariate correlations), women’s TFI was associated with lower comfort initiating sex and lower comfort refusing sex, consistent with prior findings that women’s TFI is associated with lower sexual assertiveness (Curtin et al., 2011; Greene & Faulkner, 2005). Moreover, women’s TMI was associated with lower comfort initiating sex and lower comfort refusing sex, supporting the importance of examining people’s beliefs about their partner’s gender role. Most importantly, we provided the first test of *which* of these beliefs is most important in predicting women’s sexual attitudes. When accounting for their shared variance, the effects of both TMI and TFI on women’s comfort refusing sex were reduced to non-significance. However, women’s TMI and TFI interacted to predict comfort initiating sex (see Fig. 1), such that women who were lower in their endorsement of both TMI and TFI were most comfortable initiating sex. This pattern provides novel insight by suggesting that the links between traditional ideology and women’s sexual initiation are particularly pernicious: Endorsing traditional ideology may undermine women’s comfort initiating sex even if they have relatively egalitarian beliefs about women’s gender roles but more traditional beliefs about men’s gender roles (or vice versa).

#### Men’s Traditional Beliefs

Few prior studies have examined how heterosexual men’s beliefs about men relate to their comfort initiating sex and comfort refusing sex, despite the clear theoretical and practical importance of these links. Addressing this gap, we provide the first illustration that men’s greater TMI is associated with lower comfort refusing sex but is not related to comfort initiating sex. We also demonstrate that men’s greater TFI is related to lower comfort refusing sex, highlighting the importance of examining how men’s traditional beliefs about women influence their sexual attitudes. Most importantly, however, we demonstrate *which* of these beliefs is most important to men’s sexual attitudes. In contrast to the results of initial correlations, when accounting for their shared variance, men’s TFI was no longer associated with comfort refusing sex, while greater TMI continued to be associated with lower comfort refusing sex. These results (1) suggest that men’s traditional beliefs about men may have a more robust effect on their comfort refusing sex than their beliefs about women, consistent with the strong emphasis traditional masculine roles place on men being continually ready for sex and (2) once again, emphasize the importance of accounting for both sets of beliefs simultaneously.

Intriguingly, in the opposite direction of our predictions, men’s greater TFI was associated with *lower* comfort initiating sex (with or without accounting for their TMI). Post hoc analyses offer a possible interpretation of this finding: Greater TFI was associated with men’s lower self-evaluations of knowledge and skills in sexual domains, accounting for their lower comfort initiating sex. Men with more traditional views of women as ‘pure’ and non-sexual may struggle to communicate with their partners about sex (Greene & Faulkner, 2005; Norton et al., 2016). Because sexual communication predicts women’s sexual pleasure (Jones et al., 2018), men who feel less able to sexually communicate may perceive they have insufficient skills and experience to please their partner (e.g., Oattes & Offman, 2007), thereby lowering their comfort initiating sex. Importantly, these highly speculative explanations stem from post hoc analyses and thus should be interpreted with caution until replicated.

### Limitations and Future Research Directions

The current research is the first to demonstrate the links between both men’s and women’s TMI and TFI and their comfort initiating sex and comfort refusing sex, thereby providing a novel illustration of the importance of examining both sets of beliefs simultaneously alongside their interaction. This key extension of previous work was also accomplished in a large, gender-balanced sample (women = 389, men = 393), which varied in age (*M* = 33.00, *SD* = 10.66), and the observed associations were robust controlling for factors likely to influence sexual assertiveness and traditional attitudes (i.e., self-esteem and age).

Despite these strengths, we also acknowledge the study’s limitations. Our correlational, cross-sectional data leaves open the possibility that the reverse causal direction occurs, whereby lower sexual assertiveness predicts greater endorsement of traditional gender ideology. Although this alternative proposed direction is less theoretically plausible given the social expectations of gender roles for sexual behavior, it should be further explored.

The cross-sectional nature of our data also meant that we were unable to control for prior values of the variables in our exploratory mediation analyses, limiting our ability to draw conclusions regarding the causal direction of these effects (Maxwell & Cole, 2007). However, given the strong theoretical links between men’s TFI and lower sexual knowledge and skills, we feel our proposed causal direction is more likely than the alternative. Indeed, it is more plausible that men’s relatively stable beliefs about women’s purity and non-sexual nature (e.g., Levant & Powell, 2017) undermine their ability or willingness to obtain key sexual knowledge and skills through communicating with their partner than that their lack of knowledge and skills encourages more traditional beliefs. This causal direction is also supported by the content of the traditional feminine ideology construct (Levant et al., 2007) which dictates that, not only should women be non-sexual (e.g., “It is not acceptable for a woman to masturbate”), but women should not expect to experience pleasurable sex for which their partner’s knowledge and skill would be required (e.g., “A woman should not expect to be sexually satisfied by her partner”). Nonetheless, the reverse causal direction whereby men’s lower sexual knowledge and skills increase their TFI remains possible. It is also possible that causal links operate in both directions whereby traditional beliefs undermine knowledge and skills which, in turn, encourage more traditional beliefs. Thus, we urge caution in concluding that possessing lower sexual knowledge and skills is the definitive causal mechanism underlying the link between men’s TFI and their comfort initiating sex until further analyses can confirm the causal direction of the presented effects.

As discussed previously, assessing sexual assertiveness rather than sexual behavior circumvents the influence of complex contextual factors which may govern people’s sexual behavior. However, as we assessed men’s and women’s self-reported feelings of comfort initiating and refusing sex, and not actual sexual behavior, more research is needed to ascertain the extent to which such reported comfort translates to enacted initiation and refusal within relationships (e.g., in daily life or experimental scenarios; Day et al., 2015). However, given the links between sexual assertiveness and achieving pleasurable and satisfactory sexual relationships (Apt & Hurlbert, 1993; Greene & Faulkner, 2005; Hurlbert, 1991; Morokoff et al., 1997), we expect that feelings of comfort will lead to critical downstream behaviors such as sexual consent behaviors (e.g., Darden et al., 2019) and orgasm (Lentz & Zaikman, 2021). Future research should also employ more comprehensive measures of sexual assertiveness, as we only assessed one-item measures.

Previous theoretical and empirical work suggests that the influence of traditional gender roles within sexual domains may become less pronounced in long-term intimate relationships (Byers, 1996; Masters et al., 2013; Milhausen & Herold, 1999). However, by demonstrating the associations between TMI and TFI and sexual assertiveness within long-standing (*M* = 7 years, *SD* = 7.30) relationships, our results suggest that traditional gender role beliefs may continue to influence men’s and women’s sex lives in established relationships. In sexual contexts, traditional gender roles function to provide sexual ‘scripts’ that help guide behavior and lend predictability to interactions in uncertain situations (Simon & Gagnon, 1986). Therefore, as couples become more familiar with each other and uncertainty decreases, adherence to traditional gender role-consistent behavior decreases (e.g., women initiate sex, men refuse sex; Byers 1996; Masters et al., 2013; Milhausen & Herold, 1999). Yet, these studies have not explored whether gender-discrepant sexual behaviors emerge for men and women with more traditional beliefs about gender roles. Thus, the current research provides an important extension by demonstrating that people who hold traditional beliefs about gender roles may continue to be constrained by traditional expectations, even in long-term relationships.

The current research only examined traditional gender ideology and sexual assertiveness in established relationships, and future work could extend our findings to other relevant contexts. Namely, the links between traditional gender role beliefs and sexual attitudes and behavior should be especially relevant outside established relationships, in which sexual interactions are more uncertain and sexual scripts more heavily relied upon (e.g., one-night stands). Therefore, we provided a conservative test by examining relatively established and satisfying relationships. This demographic reported generally high comfort initiating sex and high comfort refusing sex and is likely respectful of their partner’s needs to engage/not engage in sex (e.g., Muise et al., 2017). Thus, traditional ideology may particularly influence individuals’ ability to navigate uncertain sexual experiences with new partners or negotiate sexual consent and future work should explore these links.

Future work could also examine other domains of established relationships beyond sex. Within romantic relationships, traditional gender roles prescribe different behaviors for men (protect, provide, and lead the family) and women (nurture, support, and care for the family; Mahalik et al., 2003, 2005), and greater internalization of these beliefs (higher traditional gender ideology) should influence men’s and women’s relationship functioning. Yet, very few studies have examined these associations, and none have taken the current research’s approach of examining the outcomes of TMI and TFI together.

Future research should also examine dyadic effects. Our conclusions are limited in that we indirectly assessed people’s beliefs about their partner’s gender roles through their beliefs about men’s and women’s roles in general. However, it is possible that women’s general belief that men should be sexually agentic undermines their comfort initiating sex, but that their specific view of their partner’s role is less rigid, allowing them to feel more comfortable initiating sex. To address this limitation, future research could examine how traditional gender ideology relates to sexual behavior within romantic relationships (both sexual and otherwise) using dyadic methods that assess and account for both an individual’s and their partner’s traditional gender beliefs.

### Practice Implications

The associations between men’s and women’s TFI and TMI and their comfort initiating sex and comfort refusing sex suggest that, contrary to dominant perspectives, traditional gender role beliefs about both *one’s own* and *the other* set of traditional gender roles may increase harmful sexual attitudes. These attitudes may, in turn, undermine couples’ ability to achieve pleasurable and satisfactory sexual relationships (e.g., Hurlbert, 1991). Thus, when future research replicates the observed effects and establishes causality, these results may offer directions for interventions targeting men’s and women’s sexual well-being. Such initiatives should address the unique pressures and restrictions men and women place on themselves through their beliefs about both their own and others’ gender roles by (1) highlighting and confronting beliefs relevant to the self and others to exemplify the implicit presence and negative consequences of the social pressures both men and women face, (2) encouraging positive behavior which is congruent with the person’s own sexual desires, even if the behavior is incongruent with these social pressures, and (3) facilitating open conversations between intimate partners regarding each of the above topics to ensure that unspoken assumptions based on traditional beliefs do not hinder sexual well-being.

## Conclusion

Current perspectives suggest that understanding how traditional gendered beliefs influence people’s sexual attitudes requires examining only their beliefs about their own traditional gender role. Yet, people’s beliefs about the *other* set of gender roles should also have important implications for their thoughts, feelings, and behavior. The results presented in this study demonstrate the importance of accounting for both sets of beliefs together to understand people’s sexual attitudes. For instance, although a woman may hold relatively egalitarian views of either her own or men’s gender role, the observed results suggest she will only be more comfortable initiating sex if she holds neither set of traditional beliefs. Likewise, a man’s traditional beliefs about his own roles are likely to predict lower comfort refusing sex, while his beliefs about women’s gender roles will predict lower comfort initiating sex. Thus, as sexual assertiveness is important for achieving pleasurable and satisfactory sexual relationships (e.g., Hurlbert 1991), these results highlight the importance of considering both sets of traditional beliefs together for understanding the potential impact of these beliefs on people’s lives.

## Electronic supplementary material

Below is the link to the electronic supplementary material.

Supplementary Material 1

## Data Availability

Analysis code and materialsare available in the Online Supplemental Materials. Data and syntax needed to reproduce the results are available via the Open Science Framework (https://osf.io/er7fx/).

## Code Availability

NA
