# Supplementary material for: It Takes Two to Tango: Links Between Traditional Beliefs About both Men’s and Women’s Gender Roles and Comfort Initiating Sex and Comfort Refusing Sex
Source: Sex Roles. 2023 Apr 29;88(11-12):514–28. doi: 10.1007/s11199-023-01366-w (PMC10148619; doi:10.1007/s11199-023-01366-w)
Supplement: Supplementary file 1 — Supplementary Material 1 [file 11199_2023_1366_MOESM1_ESM.docx]

**Online Supplemental Materials**

**It Takes Two to Tango: Links Between Traditional Beliefs About Both Men’s and Women’s Gender Roles and Comfort Initiating Sex and Comfort Refusing Sex**

1. Selecting Items From the Full Femininity Ideology Scale 2

2. Scales and Items Used in Manuscript Analyses 4

3. Attention Checks Employed in Study 5

4. Syntax for Analyses 5

9. Syntax for Post Hoc Additional Analyses 6

**1. Selecting Items From the Full Femininity Ideology Scale**

Given our measures were part of a broader study, to reduce participant burden we generated a shorter version of the Femininity Ideology Scale (Levant et al., 2007). Unfortunately, when we administered the survey, we were unaware that a short-form version of the scale had already been validated in previous research (Levant et al., 2017). However, of the eight items employed in analyses, six appear in the shorter version of the scale (bolded items below). When administering items to participants, our approach was to enhance reliability of the shortened measure by selecting the highest loading items from each subscale that were not (1) overtly antiquated (e.g., “Women should not want to succeed in the business world because men will not want to marry them”) or (2) too specific for use in a shorter assessment (e.g., “A woman should not tell dirty jokes”, “A woman should remain a virgin until she is married;” Levant et al., 2007). For our manuscript analyses, we then decided to exclude an item that (3) directly referenced one of our outcomes, and thus was potentially recursive (i.e., “A woman should not initiate sex”; Levant et al., 2007), and (4) an item we deemed beyond the scope of the present focus from the Stereotypic Image subscale (“Women should have large breasts”). We outline the included and removed items in the table below.

| **Subscale of the Femininity Ideology Scale** (Levant et al., 2007) | **Femininity Ideology Scale Items Included in Analyses** | **Femininity Ideology Scale Items Removed from each Subscale^1^** |
| --- | --- | --- |
| **Deference/**  **Dependence** | **A woman should not expect to be sexually satisfied by her partner.**  A woman should not make more money than her partner.  **A woman’s worth should be measured by the success of her partner.**  **A woman should not consider her career as important as a man’s.** | Women should not want to succeed in the business world because men will not want to marry them.  A woman should not be competitive.  Women should have men make decisions for them.  Women should act helpless to attract a man.  A woman should not marry a younger man.  *A woman should not initiate sex. (removed for analysis)* |
| **Purity** | **Women should not view/use pornographic material.**  **It is not acceptable for a woman to masturbate.**  **A woman should not swear.**  A woman should not have a baby until she is married. | A woman should remain a virgin until she is married.  A woman should not tell dirty jokes.  A woman should be dependent on religion and spirituality for guidance.  Women should dress conservatively so they do not appear loose.  If a woman chooses to have an abortion, she should not feel guilty. |
| **Stereotypic Image** |  | *Women should have large breasts. (removed for analysis)* |

*Note.* Bolded items appear in the previously validated short-form version of the scale. ^1^Italicized items were administered to participants but removed prior to analysis. All other removed items were not administered to participants.

**2. Scales and Items Used in Manuscript Analyses**

| **Scale** | **Items** |
| --- | --- |
| **The Male Role Norms Inventory Very Brief^1^** (McDermott et al., 2018) | Men should watch football games instead of soap operas.  Boys should prefer to play with trucks rather than dolls.  A man should always be the boss.  I think a young man should try to be physically tough, even if he’s not big.  Men should not be too quick to tell others that they care about them. |
| **Selected Items from the Femininity Ideology Scale**  (Levant et al., 2007 | A woman should not expect to be sexually satisfied by her partner.  A woman should not make more money than her partner.  A woman’s worth should be measured by the success of her partner.  A woman should not consider her career as important as a man’s.  Women should not view/use pornographic material.  It is not acceptable for a woman to masturbate.  A woman should not swear.  A woman should not have a baby until she is married. |
| **Comfort Initiating Sex** | I am comfortable initiating sex |
| **Comfort Refusing Sex** | I am comfortable refusing sex |
| **The Rosenberg Self-Esteem Scale** (Rosenberg, 1965) | I feel that I’m a person of worth, at least on an equal plane with others.  I feel that I have a number of good qualities.  All in all, I am inclined to feel that I am a failure.  I am able to do things as well as most other people.  I feel I do not have much to be proud of.  I take a positive attitude toward myself.  On the whole, I am satisfied with myself.  I wish I could have more respect for myself.  I certainly feel useless at times.  At times I think I am no good at all. |
| **Sexual Knowledge & Skill** | I have the knowledge and skills needed to have a satisfying sex life. |

*Note.* ^1^As we did with the Femininity Ideology Scale (see section 1), for our analyses we removed two administered items that were directly related to our outcome measures: “*A man should not turn down sex*,” and “*A man should always be ready for sex.”*

**3. Attention Checks Employed in Study**

We excluded participants who failed the attention check in the shorter version of the survey:

“It's important that you pay attention to this study. Please select 'Strongly Disagree'.”

or any of the three attention checks in the longer version of the survey:

“It's important that you pay attention to this study. Please select 'Strongly Disagree'.”

“I am paying attention. If you are paying attention, select Strongly Disagree.”

“I am paying attention. If you are paying attention, select number two.”

**4. Syntax for Analyses**

*Note.* Initiating = comfort initiating sex, refusing = comfort refusing sex, tfi_c = traditional femininity ideology (mean centred), tmi_c = traditional masculinity ideology (mean centred).

Variables separated by an ‘x’ denote an interaction term of these variables, e.g., tfixtmi is the interaction of traditional masculinity ideology and traditional femininity ideology.

1. **Main Analyses**

**File Split by Gender**

SORT CASES BY gender.

SPLIT FILE LAYERED BY gender.

REGRESSION

/MISSING LISTWISE

/STATISTICS COEFF OUTS CI(95) R ANOVA CHANGE

/CRITERIA=PIN(.05) POUT(.10)

/NOORIGIN

/DEPENDENT initiating

/METHOD=ENTER tfi_c tmi_c tmixtfi.

REGRESSION

/MISSING LISTWISE

/STATISTICS COEFF OUTS CI(95) R ANOVA CHANGE

/CRITERIA=PIN(.05) POUT(.10)

/NOORIGIN

/DEPENDENT refusing

/METHOD=ENTER tfi_c tmi_c tmixtfi.

SPLIT FILE OFF.

**b) Hierarchical Regression Analyses Controlling for Age and Self-Esteem**

*Note.* selfesteem_c = self-esteem (mean centred), age_c = age (mean centred).

SORT CASES BY gender.

SPLIT FILE LAYERED BY gender.

REGRESSION

/MISSING LISTWISE

/STATISTICS COEFF OUTS R ANOVA CHANGE CI(95)

/CRITERIA=PIN(.05) POUT(.10)

/NOORIGIN

/DEPENDENT initiating

/METHOD=ENTER age_c selfesteem_c

/METHOD=ENTER tfi_c tmi_c tmixtfi.

REGRESSION

/MISSING LISTWISE

/STATISTICS COEFF OUTS R ANOVA CHANGE CI(95)

/CRITERIA=PIN(.05) POUT(.10)

/NOORIGIN

/DEPENDENT refusing

/METHOD=ENTER age_c selfesteem_c

/METHOD=ENTER tfi_c tmi_c tmixtfi.

SPLIT FILE OFF.

**c) Mediation Analyses Conducted in PROCESS**

To explore the unexpected negative association between men’s TFI and their comfort initiating sex, we conducted a mediation analysis on only the men sampled using the PROCESS macro for SPSS 26 (model 4, estimating 10,000 bootstrap resamples; Hayes, 2017). We tested whether the association between men’s TFI and comfort initiating sex was mediated by their perception of their skills and knowledge about sex, while simultaneously accounting their TMI as a covariate. The following syntax illustrates the model used to conduct these analyses. Uppercase represents a variable and lowercase represents required PROCESS syntax.

process y=INITIATING/m=KNOWLEDGESKILLS/x=TFI/bmatrix=1,1,1/ cov=TMI.
